# Unraveling the causality between gastroesophageal reflux disease and increased cancer risk: evidence from the UK Biobank and GWAS consortia

**DOI:** 10.1186/s12916-024-03526-5

**Published:** 2024-08-07

**Authors:** Gujie Wu, Yaqiong Liu, Dong Ning, Mengnan Zhao, Xiaoqing Li, Lu Chang, Qili Hu, Yao Li, Lin Cheng, Yiwei Huang

**Affiliations:** 1 Department of Thoracic Surgery, Zhongshan Hospital, Fudan University, Shanghai, 200032 China; 2 Endoscopy Center and Endoscopy Research Institute, Zhongshan Hospital, Fudan University, Shanghai, China; 3 Regenerative Medicine Institute, School of Medicine, National University of Ireland (NUI), Galway, Ireland; 4 Department of Physiology, School of Medicine, National University of Ireland (NUI), Galway, Ireland; 5 Department of Laboratory Medicine, Huashan Hospital, Fudan University, Shanghai, China; 6 Department of Radiology, Huashan Hospital, State Key Laboratory of Medical Neurobiology, Fudan University, Shanghai, China

**Keywords:** Gastroesophageal reflux disease, Cancer risk, Mendelian randomization, Genome-wide association studies, Lifestyle factors

## Abstract

**Background:**

Gastroesophageal reflux disease (GERD) is a common condition characterized by the reflux of stomach contents into the esophagus. Despite its widespread prevalence worldwide, the causal link between GERD and various cancer risks has not been fully established, and past medical research has often underestimated or overlooked this relationship.

**Methods:**

This study performed Mendelian randomization (MR) to investigate the causal relationship between GERD and 19 different cancers. We leveraged data from 129,080 GERD patients and 473,524 controls, along with cancer-related data, obtained from the UK Biobank and various Genome-Wide Association Studies (GWAS) consortia. Single nucleotide polymorphisms (SNPs) associated with GERD were used as instrumental variables, utilizing methods such as inverse variance weighting, weighted median, and MR-Egger to address potential pleiotropy and confounding factors.

**Results:**

GERD was significantly associated with higher risks of nine types of cancer. Even after adjusting for all known risk factors—including smoking, alcohol consumption, major depression, and body mass index (BMI)—these associations remained significant, with higher risks for most cancers. For example, the adjusted risk for overall lung cancer was (OR, 1.23; 95% CI: 1.14–1.33), for lung adenocarcinoma was (OR, 1.18; 95% CI: 1.03–1.36), for lung squamous cell carcinoma was (OR, 1.35; 95% CI: 1.19–1.53), and for oral cavity and pharyngeal cancer was (OR, 1.73; 95% CI: 1.22–2.44). Especially noteworthy, the risk for esophageal cancer increased to (OR, 2.57; 95% CI: 1.23–5.37). Mediation analyses further highlighted GERD as a significant mediator in the relationships between BMI, smoking, major depression, and cancer risks.

**Conclusions:**

This study identifies a significant causal relationship between GERD and increased cancer risk, highlighting its role in cancer development and underscoring the necessity of incorporating GERD management into cancer prevention strategies.

**Supplementary Information:**

The online version contains supplementary material available at 10.1186/s12916-024-03526-5.

## Background

Gastroesophageal reflux disease (GERD) is a chronic gastrointestinal disorder characterized by the backflow of stomach contents into the esophagus, affecting millions of people globally [1]. This condition leads to symptoms such as heartburn, acid regurgitation, and chest pain, which significantly impair quality of life by causing sleep disturbances, reduced work productivity, and limitations in daily activities [2]. Currently, up to 20% of individuals in Western populations experience GERD symptoms on a weekly basis, and the rising trend of GERD has garnered increasing attention [1, 2].

Recent epidemiological research has identified a potential link between GERD and an increased risk of multiple cancers [3–7]. These associations may be due to the damaging effects of gastric acid and bile on mucosal surfaces beyond the esophagus, suggesting a need for a thorough investigation into GERD’s broader oncogenic potential [8]. However, existing observational studies, while highlighting a potential link, have failed to definitively establish a causal relationship between GERD and cancer. This gap is partly attributable to confounding factors such as smoking, alcohol use, obesity, and major depression, which are known risk factors for both GERD and various cancers [9]. Therefore, determining the causal relationship between GERD and cancer requires a methodology capable of addressing these complexities [10].

Mendelian randomization (MR) offers a robust method to explore causal relationships by using genetic variants as proxies for modifiable risk factors, thereby minimizing confounding and reverse causation [11]. The advent of comprehensive genome-wide association studies (GWAS) has enhanced the feasibility of MR to investigate GERD’s causal effects on cancer risk [12]. Despite these advancements, prior MR studies have primarily focused on cancers localized to the gastrointestinal tract, without thoroughly examining systemic cancers and the influence of lifestyle factors such as smoking, alcohol consumption, body mass index (BMI), and major depression [13].

Our study aims to address these gaps by using MR to explore the causal relationship between GERD and both proximal and systemic cancer risks. Specifically, we extend the investigation beyond proximal organ cancers to include a broader range of systemic impacts, while adjusting for lifestyle and mental health factors such as smoking, alcohol use, BMI, and major depression. By integrating data from multiple GWAS and employing rigorous statistical methods, our study seeks to provide a comprehensive understanding of the potential causal links between GERD and various cancer types. Furthermore, our research highlights the importance of integrating GERD management into cancer prevention strategies. By establishing a clearer causal relationship, our findings could influence future screening recommendations, guide lifestyle and dietary advice for GERD patients, and inform clinical practices regarding GERD management with an oncogenic perspective.

## Method

### Study design and overview

**Fig. 1:**
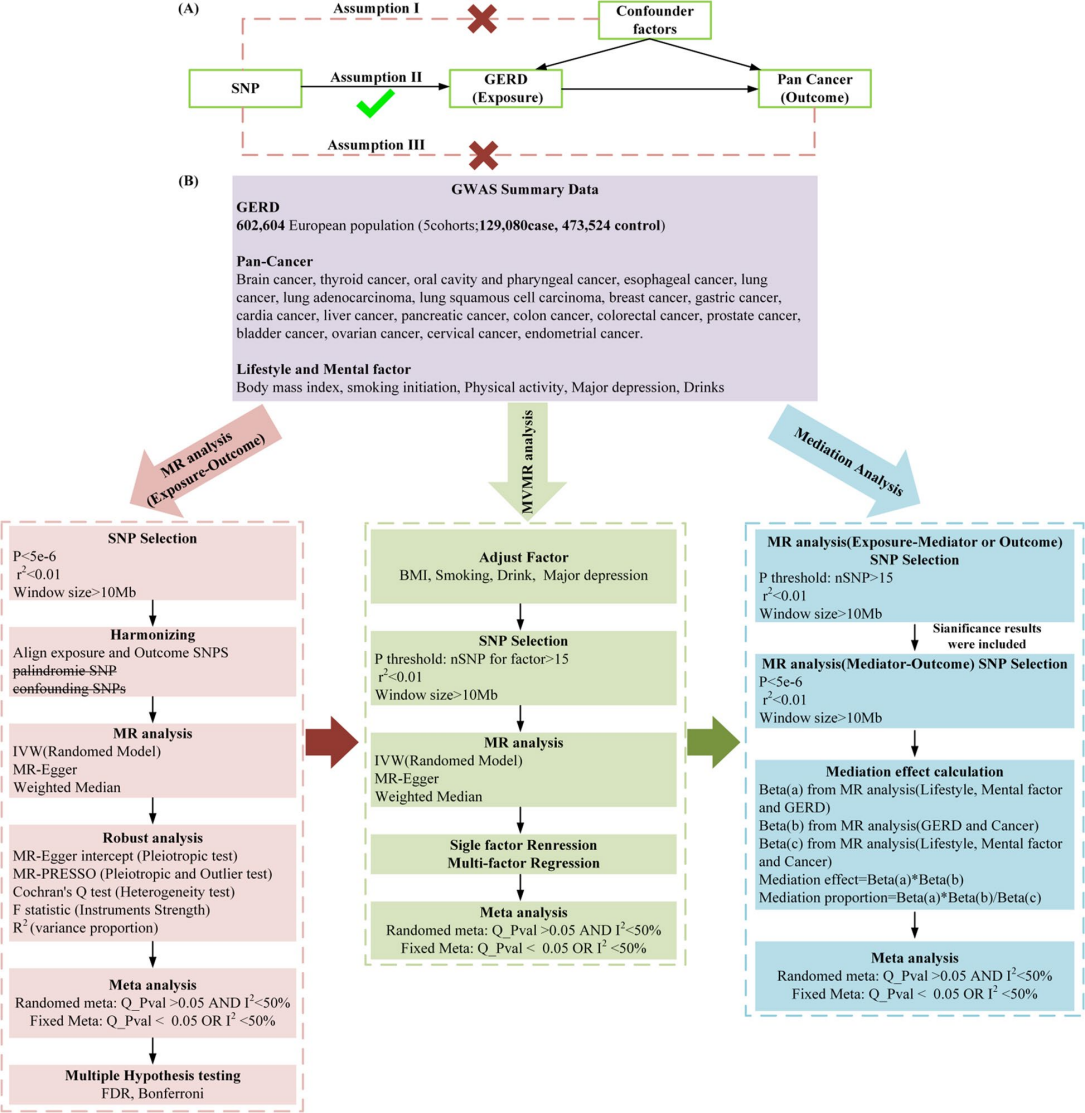
MR assumption and flow chart. **A** MR assumption: I, SNPs are not associated with confounder; II, SNPs are strongly associated with the GERD; III, SNPs influence the cancer solely through the GERD. **B** Analysis flow chart

In our study, we adopted a two-sample MR approach to elucidate the causal relationships between GERD and the risk of various cancers. This investigation further employed a multivariable MR technique to discern the direct effects of GERD on cancer risk. We also identified a range of mediators that influence the connection between GERD and cancer risk across different cancer types. The comprehensive design of our research is illustrated in Fig. 1. Our analysis drew upon publicly available summary statistics related to GERD, potential mediators, and pan-cancer outcomes from previous publications and consortia. As these primary studies had secured approval from their respective Institutional Review Boards (IRBs), our secondary analysis did not necessitate additional IRB consent.

### Data sources of exposure and outcome

The exposure dataset for GERD was developed by Ong et al. [14]. This work implemented a multitrait GWAS model that integrated GWAS for BMI, major depressive disorder (MDD), educational attainment, GERD, and Barrett’s esophagus (BE) to unveil additional susceptibility loci for GERD. This comprehensive dataset included 129,080 cases and 473,524 controls of European ancestry, directly retrieved from the GWAS Catalog (https://www.ebi.ac.uk/gwas/studies/GCST90000514). The outcome datasets covered a wide array of cancers, including brain cancer, thyroid cancer, oral cavity and pharyngeal cancer, esophageal cancer, lung cancer, lung adenocarcinoma, lung squamous cell carcinoma, breast cancer, gastric cancer, cardia cancer, liver cancer, pancreatic cancer, colon cancer, colorectal cancer, prostate cancer, bladder cancer, ovarian cancer, cervical cancer, and endometrial cancer. These datasets were acquired from the UK Biobank and a variety of other GWAS consortia, including ILCCO, TRICL-UK, FinnGen, OncoArray Consortium, OCAC, Schumacher FR et al., BCAC, and O’Mara TA et al. Similarly, mediator variables were sourced from various datasets provided by the GIANT Consortium, GSCAN Consortium, Within-family GWAS Consortium, PGC Consortium, and the GWAS and Sequencing Consortium of Alcohol and Nicotine use. For a comprehensive overview of these data sources and their access links, refer to Additional file 1: Table S1.

### SNP selection

Our MR analysis aimed to investigate potential causal connections between GERD and a spectrum of cancers using genetic variants as instrumental variables (IVs). The validity of an MR analysis rests on three critical assumptions: (1) IVs are not confounded by external factors; (2) there is a strong association between IVs and the exposure; and (3) IVs influence the outcome solely through the exposure [15]. Initially, we selected single nucleotide polymorphisms (SNPs) exhibiting a genome-wide significant association (*P* < 5 × 10^−8) with the traits of interest as IVs. When necessary, we expanded our selection to include SNPs with *P* values up to 5e−6 to ensure a robust MR analysis. Linkage disequilibrium clumping was then performed to exclude closely linked SNPs (*r*^2 < 0.01, window size > 10 MB) [16]. Palindromic SNPs with allele frequencies near 0.5 were also excluded to synchronize the exposure and outcome datasets. The list of selected SNPs used in the study can be found in Additional file 1: Table S2. The effectiveness of our genetic instruments was validated by calculating the *F* statistic [17], ensuring a minimal risk of weak instrument bias [18].

### Statistical analysis

Our primary analysis was conducted using the inverse variance-weighted (IVW) method [19], renowned for its efficacy in establishing causal inferences. Supplemental analyses included the weighted median approach [20] and MR-Egger regression [21] to account for potential pleiotropy. The MR-PRESSO test [22] was utilized to detect pleiotropy and outliers, with Cochran’s *Q* test assessing heterogeneity [23]. For significant heterogeneity, a random-effects IVW model was applied. To mitigate the risk of false discoveries due to multiple testing, we employed the false discovery rate (FDR) [24] and Bonferroni correction [25]. Analyses were performed using the two-sample MR, Mendelian randomization, and MR-PRESSO packages in R (version 4.2.1). Mediation analysis employed a two-step MR approach to evaluate indirect effects, with mediation effect size and significance assessed via the product of coefficients method [26] and Sobel test [27].

## Results

### Causal effect analysis of GERD on site-specific cancers

**Fig. 2:**
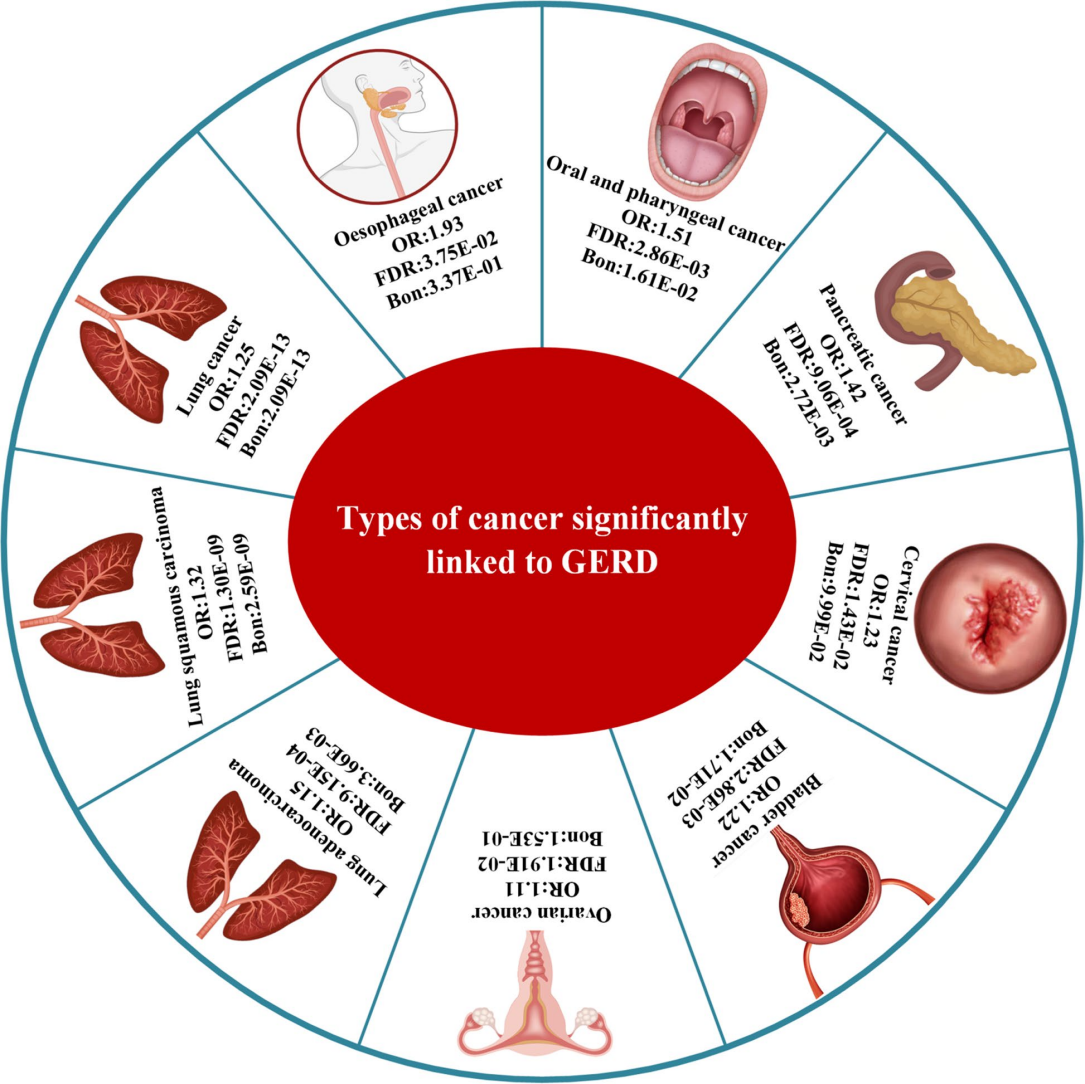
Simplified schematics of GERD's effects on various cancers

Our rigorous exploration of the causal effects of GERD on the incidence of various cancers at specific sites integrated genetic data from extensive GWAS across multiple databases, comprehensively assessing the links between GERD and 19 different cancer sites. In our methodological approach, we utilized MR to infer causation using genetic variations as instrumental variables, significantly reducing the confounding often seen in observational studies. The MR analysis unveiled associations between GERD and various types of cancer, as itemized in Additional file 1: Table S3. To avoid the influence of SNP confounders, we used LD traits [28] to identify 63 unique potential confounding SNPs, as shown in Additional file 1: Table S4. After removing these confounding SNPs, we re-evaluated the association between GERD and various cancers across multiple databases using MR, retained the valid results, and conducted a meta-analysis on these results (Table 1). Post-application of false discovery rate (FDR) correction, the meta-analysis results indicated a significant association between GERD and increased risk of various cancers (Fig. 2). Specifically, GERD was associated with higher incidence rates of lung cancer (OR = 1.25, 95% CI: 1.18–1.32), lung adenocarcinoma (OR = 1.15, 95% CI: 1.07–1.23), lung squamous carcinoma (OR = 1.32, 95% CI: 1.21–1.44), bladder cancer (OR = 1.22, 95% CI: 1.08–1.36), ovarian cancer (OR = 1.11, 95% CI: 1.03–1.20), and pancreatic cancer (OR = 1.42, 95% CI: 1.19–1.71). Additionally, results from individual datasets indicated significant associations between GERD and cervical cancer in the FinnGen dataset (OR = 1.23, 95% CI: 1.06–1.42), esophageal cancer in the UK Biobank dataset (OR = 1.93, 95% CI: 1.12–3.35), and oral and pharyngeal cancer in the Lesseur dataset (OR = 1.51, 95% CI: 1.18–1.93). There was no significant association between GERD and other types of cancer.

**Table 1:** MR analysis of GERD association with multiple cancer risks

**Outcome**	**Number of SNPs**	**OR(95% CI)**	***P* val**	**FDR**	**Bonferroni**
**Brain cancer (FinnGen)**	196	0.95(0.72–1.26)	0.742		
**Brain cancer (UK Biobank)**	199	1.14(0.81–1.61)	0.449		
**Meta (Q_Pval = 0.426, *I*^2 = 0%)**		1.03(0.82–1.27)	0.824	8.73E−01	1.00E+00
**Thyroid cancer (FinnGen)**	196	0.95(0.77–1.16)	0.583		
**Thyroid cancer (UK Biobank)**	199	1.3(0.92–1.83)	0.133		
**Meta (Q_Pval = 0.116, *I*^2 = 60%)**		1.07(0.79–1.46)	0.646	0.776	1.00E+00
**Oral cavity and pharyngeal cancer (Lesseur)**	170	1.51(1.18–1.93)	0.001	2.86E−03	1.61E−02
**Esophageal cancer (UK Biobank)**	199	1.93(1.12–3.35)	0.019	3.75E−02	3.37E−01
**Lung cancer (UK Biobank)**	199	1.58(1.14–2.21)	0.007		
**Lung cancer (ILCCO)**	192	1.27(1.14–1.41)	<0.001		
**Lung cancer (TRICL)**	198	1.24(1.15–1.34)	<0.001		
**Lung cancer (FinnGen)**	196	1.18(1.03–1.36)	0.019		
**Meta (Q_Pval = 0.458, *I*^2 = 0%)**		1.25(1.18–1.32)	<0.001	2.09E−13	2.09E−13
**Lung cancer (adenocarcinoma_FinnGen)**	196	1.19(0.95–1.49)	0.123		
**Lung cancer (adenocarcinoma_ILCCO)**	192	1.12(0.96–1.3)	0.147		
**Lung cancer (adenocarcinoma_TRICL)**	199	1.15(1.05–1.26)	0.002		
**Meta (Q_Pval = 0.892, *I*^2 = 0%)**		1.15(1.07–1.23)	<0.001	9.15E−04	3.66E−03
**Lung cancer (squamous_FinnGen)**	196	1.2(0.93–1.54)	0.152		
**Lung cancer (squamous_ILCCO)**	192	1.32(1.14–1.54)	<0.001		
**Lung cancer (squamous_TRICL)**	199	1.35(1.2–1.51)	<0.001		
**Meta (Q_Pval = 0.719, *I*^2 = 0%)**		1.32(1.21–1.44)	<0.001	1.30E−09	2.59E−09
**Breast cancer (BCAC)**	190	1.01(0.96–1.07)	0.676		
**Breast cancer (FinnGen)**	196	0.97(0.9–1.04)	0.428		
**Breast cancer (UK Biobank)**	199	0.94(0.56–1.6)	0.824		
**Meta (Q_Pval = 0.660, *I*^2 = 0%)**		1.00(0.96–1.04)	0.884	8.84E−01	1.00E+00
**Gastric cancer (FinnGen)**	196	1.08(0.87–1.36)	0.478		
**Gastric cancer (UK Biobank)**	199	1.4(1.02–1.94)	0.04		
**Meta (Q_Pval = 0.200, *I*^2 = 39%)**		1.18(0.98–1.42)	0.079	1.19E−01	1.00E+00
**Cardia cancer (UK Biobank)**	199	1.11(0.63–1.93)	0.725	8.15E−01	1.00E+00
**Pancreatic cancer (FinnGen)**	196	1.43(1.15–1.79)	0.002		
**Pancreatic cancer (UK Biobank)**	199	1.4(1.02–1.92)	0.038		
**Meta (Q_Pval = 0.905, *I*^2 = 0%)**		1.42(1.19–1.71)	<0.001	9.06E−04	2.72E−03
**Colon cancer (FinnGen)**	196	0.81(0.71–0.91)	0.001		
**Colon cancer (UK Biobank)**	199	1(0.86–1.16)	0.998		
**Meta (Q_Pval = 0.030, *I*^2 = 79%)**		0.89(0.72–1.10)	0.301	4.16E−01	1.00E+00
**Colorectal cancer (FinnGen).gz**	196	0.89(0.8–0.99)	0.035		
**Colorectal cancer (UK Biobank)**	199	0.97(0.72–1.32)	0.854		
**Meta (Q_Pval = 0.595, *I*^2 = 0%)**		0.90(0.81–1.00)	0.041	6.72E−02	7.39E−01
**Prostate cancer (FinnGen)**	196	1.02(0.94–1.11)	0.587		
**Prostate cancer (UK Biobank)**	199	0.96(0.88–1.06)	0.435		
**Prostate cancer (Schumacher FR)**	197	0.94(0.89–0.98)	0.01		
**Meta (Q_Pval = 0.193, *I*^2 = 39%)**		0.96(0.92–1.00)	0.037	6.72E−02	6.72E−01
**Bladder cancer (FinnGen)**	196	1.19(1.01–1.41)	0.034		
**Bladder cancer (UK Biobank)**	199	1.24(1.05–1.45)	0.011		
**Meta (Q_Pval = 0.776, *I*^2 = 0%)**		1.22(1.08–1.36)	0.001	2.86E−03	1.71E−02
**Ovarian cancer (FinnGen)**	196	1.07(0.86–1.33)	0.556		
**Ovarian cancer (UK Biobank)**	199	1.17(0.91–1.51)	0.21		
**Ovarian cancer (OCAC)**	190	1.11(1.02–1.21)	0.021		
**Meta (Q_Pval = 0.860, *I*^2 = 0%)**		1.11(1.03–1.20)	0.009	1.91E−02	1.53E−01
**Cervical cancer (FinnGen)**	196	1.23(1.06–1.42)	0.006	1.43E−02	9.99E−02
**Endometrial cancer (O’Mara TA)**	199	1.19(1.09–1.29)	<0.001		
**Endometrial cancer (UK Biobank)**	199	0.91(0.7–1.17)	0.443		
**Meta (Q_Pval = 0.048, *I*^2 = 74%)**		1.07(0.82–1.38)	0.632	7.76E−01	1.00E+00

FDR: method to correct *P* values for multiple comparisons; Bonferroni: adjusts *P* values for multiple comparisons to ensure stricter significance

The robustness of these findings was further validated by supplementary analyses using the weighted median (WM) and MR-Egger methods (Additional file 1: Table S5), despite some outcomes showing broad confidence intervals. The MR-PRESSO analysis (Additional file 1: Table S6) identified outliers in the datasets for lung and ovarian cancers. However, even with outlier exclusion, the associations between GERD and cancer risk retained significance. Additionally, in reverse MR analysis, we did not find a significant effect of cancer on GERD (Additional file 1: Table S7). These findings strengthen the evidence for GERD’s role in cancer etiology and underscore the need for focused prevention strategies targeting GERD.

### Multivariable MR analysis

Recognizing that smoking, alcohol consumption, obesity, and major depressive disorder are established risk factors for various cancers, we conducted a multivariable MR study to systematically mitigate these confounding effects. Our objective was to refine the direct causal relationship between GERD and overall cancer risk. Significant findings from our database were analyzed (Additional file 1: Table 8) and followed by a comprehensive meta-analysis (Table 2). Our results indicate substantial variation in the association between GERD and various cancer types when adjusting for individual or combined factors. Specifically, the link between GERD and cervical cancer was significantly influenced by adjustments for smoking habits. No significant correlation was found between GERD and the incidence of bladder or ovarian cancer after adjusting for BMI. The weekly amount of alcohol consumption did not alter the relationship between GERD and the cancer types studied. After adjusting for major depressive disorder, no significant association was observed between GERD and either bladder or cervical cancer.

**Table 2:** MR analysis between GERD and cancers adjusted for risk factors

**Outcome**	**Adjust BMI**		**Adjust drinks**		**Adjust major depression**		**Adjust smoking**		**Adjust All**	
	**OR(95% CI)**	***P*val**	**OR(95% CI)**	***P*val**	**OR(95% CI)**	***P*val**	**OR(95% CI)**	***P*val**	**OR(95% CI)**	***P*val**
**Bladder cancer**	1.18(0.93–1.49)	0.165518502	1.23(1.12–1.36)	2.99E−05	1.15(0.98–1.34)	0.081850817	1.19(1.06–1.33)	0.002374946	1.16(0.92–1.47)	0.216387267
**Cervical cancer**	1.21(1.03–1.42)	0.020287784	1.23(1.07–1.40)	0.002523341	1.17(0.94–1.44)	0.152073528	1.15(0.98–1.35)	0.088628315	1.22(0.99–1.51)	0.063601433
**Lung cancer**	1.34(1.26–1.42)	4.80E−23	1.29(1.23–1.36)	9.60E−23	1.34(1.24–1.44)	1.46E−13	1.20(1.13–1.27)	1.29E−10	1.23(1.14–1.33)	9.27E−08
**Lung cancer (adenocarcinoma)**	1.24(1.12–1.37)	4.54E−05	1.18(1.08–1.29)	0.000163614	1.21(1.07–1.37)	0.003046497	1.15(1.06–1.24)	0.000978048	1.18(1.03–1.36)	0.014669644
**Lung cancer (squamous)**	1.43(1.30–1.57)	2.04E−13	1.41(1.30–1.53)	1.32E−16	1.56(1.38–1.77)	9.24E−13	1.30(1.19–1.43)	1.29E−08	1.35(1.19–1.53)	3.76E−06
**Esophageal cancer**	2.16(1.23–3.79)	0.007204945	1.70(1.05–2.75)	0.03103533	2.13(1.08–4.22)	0.029864474	1.93(1.10–3.36)	0.020939446	2.57(1.23–5.37)	0.012027574
**Oral cavity and pharyngeal cancer**	1.90(1.46–2.47)	1.43E−06	1.56(1.25–1.94)	7.13E−05	1.46(1.05–2.03)	0.024816359	1.34(1.03–1.73)	0.027299163	1.73(1.22–2.44)	0.00191425
**Ovarian cancer**	1.07(0.99–1.17)	0.105112831	1.11(1.03–1.19)	0.00442558	1.18(1.06–1.31)	0.002663911	1.10(1.01–1.18)	0.019268421	1.06(0.95–1.18)	0.331160306
**Pancreatic cancer**	1.22(1.01–1.48)	0.037609315	1.39(1.20–1.62)	2.07E−05	1.46(1.16–1.83)	0.001032751	1.47(1.23–1.76)	1.67E−05	1.04(0.81–1.33)	0.759557172

OR(95% CI) represents the GERD’s meta effect and confidence interval on cancer after adjusting risk factor. *P* value represents the significance of meta effect

Even after adjusting for these confounding factors, significant associations between GERD and certain specific cancers (lung cancer, including adenocarcinoma and squamous cell carcinoma, oral cavity, pharyngeal cancer, and esophageal cancer) remained. Notably, most odds ratios (ORs) increased. For instance, the OR for lung cancer increased from 1.25 (95% CI: 1.18–1.32) to 1.34 (95% CI: 1.26–1.42) after adjusting for BMI, to 1.29 (95% CI: 1.23–1.36) after adjusting for alcohol intake, and to 1.34 (95% CI: 1.24–1.44) after adjusting for major depressive disorder. Similarly, the OR for lung adenocarcinoma rose from 1.15 (95% CI: 1.07–1.23) to 1.24 (95% CI: 1.12–1.37) after adjusting for BMI and to 1.21 (95% CI: 1.07–1.37) after adjusting for major depressive disorder. The OR for lung squamous cell carcinoma increased from 1.32 (95% CI: 1.21–1.44) to 1.43 (95% CI: 1.30–1.57) after adjusting for BMI, to 1.41 (95% CI: 1.30–1.53) after adjusting for alcohol intake, and to 1.56 (95% CI: 1.38–1.77) after adjusting for major depressive disorder. For esophageal cancer, the OR increased from 1.93 (95% CI: 1.10–3.35) to 2.16 (95% CI: 1.23–3.79) after adjusting for BMI and to 2.13 (95% CI: 1.08–4.22) after adjusting for major depressive disorder. The OR for oral cavity and pharyngeal cancer increased from 1.51 (95% CI: 1.18–1.93) to 1.90 (95% CI: 1.46–2.47) after adjusting for BMI and to 1.56 (95% CI: 1.25–1.94) after adjusting for alcohol intake. When all confounding factors were adjusted simultaneously, these associations remained significant, and most risks even increased. For example, the adjusted odds ratio (OR) for lung cancer was 1.23 (95% CI: 1.14–1.33), for lung adenocarcinoma was 1.18 (95% CI: 1.03–1.36), and for lung squamous cell carcinoma was 1.35 (95% CI: 1.19–1.53). The OR for oral cavity and pharyngeal cancer was 1.73 (95% CI: 1.22–2.44), and particularly noteworthy, the OR for esophageal cancer increased to 2.57 (95% CI: 1.23–5.37). These results underscore the robustness of these associations, indicating that GERD is a significant risk factor for these cancers.

### Mediation effects of GERD between lifestyle and mental health factors and various types of cancers

**Fig. 3:**
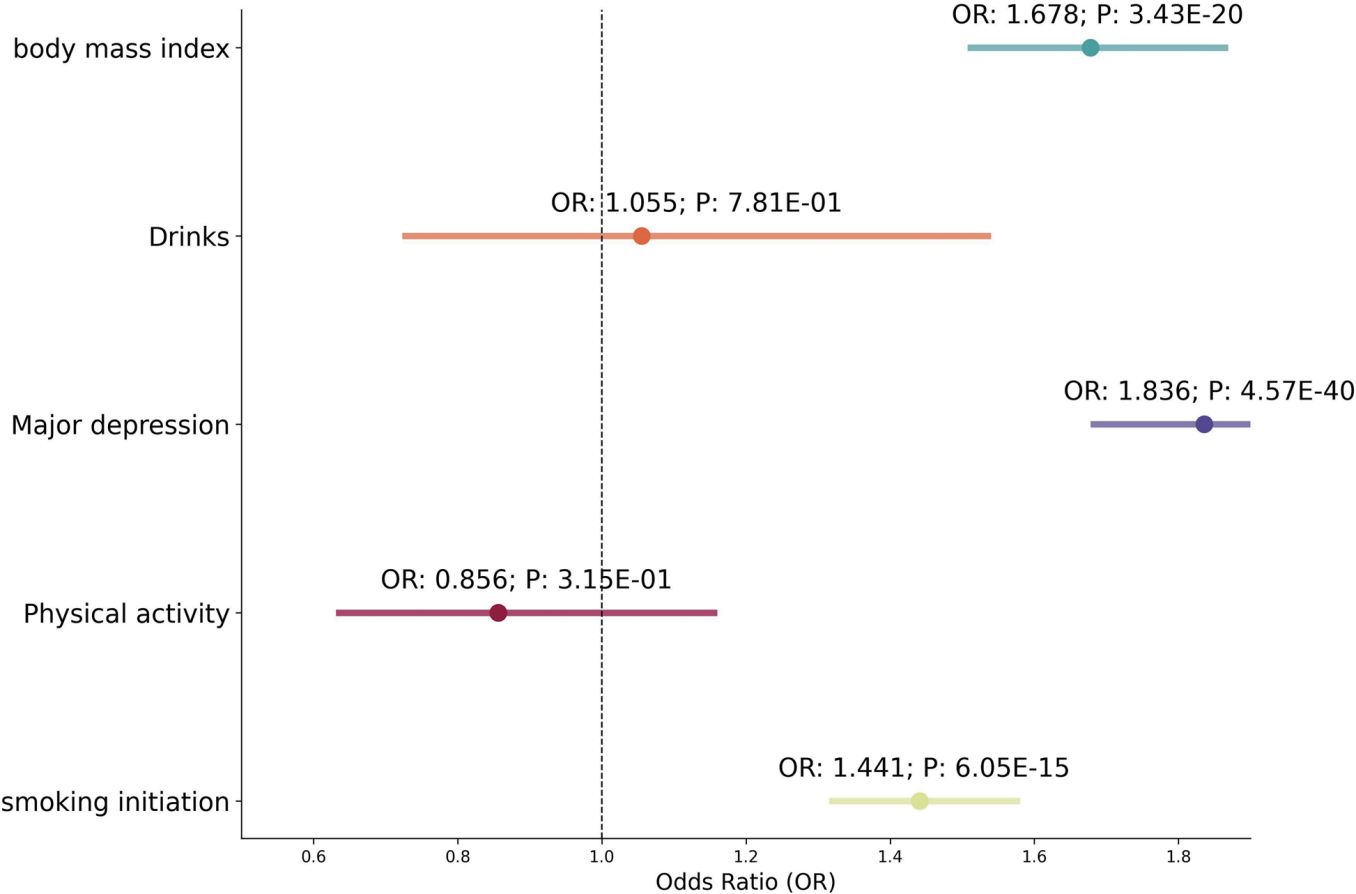
Mendelian analysis between the GERD and mediator

In our mediation analysis, we aimed to determine whether GERD serves as a mediating factor in the relationship between lifestyle and mental health factors and the risk of various cancers. Preliminary assessments evaluated the potential impact of these factors on GERD. The results of the Mendelian randomization analysis (Fig. 3) revealed significant associations between BMI, major depression, and smoking behavior with GERD. Specifically, BMI was significantly associated with GERD, with an OR of 1.68 (95% CI: 1.51–1.87), indicating that higher BMI substantially increases the likelihood of GERD. Similarly, major depression showed a significant relationship with GERD (OR = 1.84, 95% CI: 1.68–2.01), suggesting that individuals with severe depression are more prone to experiencing GERD. Additionally, smoking behavior was significantly associated with GERD (OR = 1.44, 95% CI: 1.31–1.58), indicating that smoking similarly elevates the risk of GERD. Further analysis using Mendelian randomization explored the relationships between BMI, major depression, and smoking behavior with various cancers (Additional file 1: Table S9). The results indicate that major depression is associated with an increased incidence of bladder cancer, and BMI is linked to multiple cancers, including various forms of lung cancer, bladder cancer, esophageal cancer, and pancreatic cancer. Additionally, smoking behavior is associated not only with an increased incidence of various forms of lung cancer but also with oral cancer, pharyngeal cancer, and ovarian cancer.

Table 3 and Additional file 1: Table S10 highlight the mediating roles of GERD between various exposure factors and different cancer outcomes. The analysis found that GERD mediates 28.54% of the association between severe depression and bladder cancer, underscoring a significant intermediary role of GERD. Furthermore, GERD notably mediates the relationship between BMI and various cancers, particularly in lung cancer (60.16%), lung squamous cancer (average 42.85%), and bladder cancer (44.48%). GERD also significantly mediates the impact of smoking behavior on various cancers, including lung cancer (17.98%), lung adenocarcinoma (13.60%), lung squamous cancer (12.87%), oral cancer and pharyngeal cancer (33.59%), and esophageal cancer (24.17%). These findings emphasize the complex intermediary role of GERD in the interactions between lifestyle and mental health factors and cancer risk, highlighting GERD as a significant factor in cancer prevention strategies. The observed mediation effects underscore GERD as an important risk factor for specific cancers and suggest that addressing GERD could be a beneficial component of comprehensive cancer prevention and monitoring protocols.

**Table 3:** Mediation proportion of GERD

**Exposure**	**Mediator**	**Outcome**	**Mediation proportion**
Major depression	GERD	Bladder cancer	28.54%
Body mass index	GERD	Bladder cancer	44.48%
Body mass index	GERD	Lung cancer	60.16%
Body mass index	GERD	Lung squamous cancer	42.85%
Smoking initiation	GERD	Lung cancer	17.98%
Smoking initiation	GERD	Lung adenocarcinoma	13.60%
Smoking initiation	GERD	Lung squamous cancer	12.87%
Smoking initiation	GERD	Oral cavity and pharyngeal cancer	33.59%
Smoking initiation	GERD	Esophageal cancer	24.17%

## Discussion

The role of GERD in the development of various cancers has been increasingly recognized, although direct causal relationships have yet to be definitively established [1, 2]. In this study, we employed MR to investigate the potential causal relationships between GERD and various cancer types. Our findings indicate a significant positive correlation between GERD and the risk of several cancers, including overall lung cancer, lung adenocarcinoma, lung squamous cell carcinoma, esophageal cancer, oral cavity, pharyngeal cancer, pancreatic cancer, bladder cancer, cervical cancer, and ovarian cancer.

Specifically, GERD is associated with an increased risk of lung, oral, throat, and esophageal cancers [3–7]. These observations are consistent with prior epidemiological studies suggesting that the harmful effects of stomach acids and bile, which are components of gastric contents, on various tissues could be a potential mechanism [29]. A meta-analysis revealed that individuals experiencing GERD symptoms at least weekly have nearly a fivefold increased risk of developing esophageal adenocarcinoma. Furthermore, for those with daily symptoms, this risk escalates to a sevenfold increase [3]. While our study confirms a significant association between GERD and increased cancer risks, elucidating the biological mechanisms underlying these relationships is essential. Chronic exposure to stomach acid and bile, which is common in GERD, can lead to continuous inflammation and cellular damage in the esophagus and other tissues, potentially resulting in carcinogenesis. For instance, the reflux of gastric contents into the esophagus can induce Barrett’s esophagus, a precursor to esophageal adenocarcinoma, through repeated cycles of injury and repair causing cellular changes [30, 31]. Additionally, the aspiration of acidic gastric contents into the lungs might lead to chronic inflammation and an increased risk of lung cancer, a hypothesis supported by multiple studies. Chronic inflammation in lung tissues caused by persistent exposure to gastric acid can lead to DNA damage and promote carcinogenesis. This association has been corroborated by numerous studies, collectively indicating an increased risk of lung cancer among individuals with frequent GERD symptoms [32–34]. Despite variations in methodologies and patient populations, these studies consistently demonstrate a positive correlation, suggesting that GERD may contribute to lung cancer development. Furthermore, substantial cross-disciplinary research has consistently established a significant relationship between GERD and increased risks of oral and throat cancers. Extensive research over many years, including case-control studies, cohort studies, and systematic reviews, suggests that chronic exposure to stomach acid and other refluxates due to GERD may cause inflammation and tissue damage in the oral and throat regions, thereby increasing cancer risk. These findings affirm that GERD may indeed be a contributory factor in the development of these types of cancers [4, 35–37]. Notably, our study employed both MR analysis and multivariable MR analysis to provide robust evidence of the correlation between GERD and the increased risk of lung, oral, throat, and esophageal cancers. The multivariable MR analysis, which adjusts for confounding factors such as BMI, smoking, alcohol consumption, and major depressive disorder, further strengthens the robustness of our findings. For instance, after adjusting for BMI, the ORs for various cancers, including lung cancer, increased significantly, highlighting the persistent association even after controlling for major confounders. Moreover, the OR for esophageal cancer increased substantially after adjusting for all confounding factors, underscoring the significant risk posed by GERD. These results emphasize the critical role of GERD as a significant risk factor for these cancers and highlight the need to include GERD management in cancer prevention strategies.

Currently, there is insufficient scientific evidence to directly support the correlation between GERD and the risks of pancreatic, bladder, cervical, and ovarian cancers. However, several studies have preliminarily explored the potential links between GERD and these cancers. For example, Heather Katz and colleagues reported a patient with a history of GERD who was diagnosed with esophageal adenocarcinoma at the age of 49. Shortly thereafter, metastatic lesions were discovered in her bladder. Notably, immunohistochemical staining of her bladder tumor was consistent with metastatic esophageal cancer, although clinical cases of esophageal adenocarcinoma metastasizing to the bladder are extremely rare [38]. On the other hand, Ati Burassakarn and colleagues found a significant association between a history of GERD and the prevalence of HPV infection. Given that HPV infection is widely recognized as a major risk factor for cervical cancer, this finding provides further clues for exploring the relationship between GERD and cervical cancer risk [39]. Additionally, Dana Meredith Chase and colleagues found that women diagnosed with ovarian cancer experienced symptoms of heartburn and acid reflux several months before diagnosis [40]. A study by Julia Hippisley-Cox also indicated that heartburn is independently associated with an increased risk of pancreatic cancer, with women experiencing heartburn having a 2.5-fold increased risk of developing pancreatic cancer [41]. Although the specific mechanisms by which GERD may increase the risks of these cancers remain unclear, the aforementioned studies and our current findings provide important theoretical foundations and research directions for future investigations into the relationship between GERD and increased cancer risks. These relationships likely involve complex biological mechanisms and multiple risk factors, necessitating further prospective studies and large cohort studies to validate and elucidate them.

Our mediation analysis suggests that GERD may serve as an important mediator in the relationships between BMI, major depression, smoking, and various cancers. The relationship between BMI and GERD has been extensively studied. A high BMI increases abdominal pressure, promoting the reflux of stomach contents into the esophagus, thereby leading to GERD [42, 43]. The relationship between major depression and GERD has also been thoroughly investigated. Patients with depression often experience gastrointestinal symptoms, including GERD [44]. Studies indicate that depression might increase the risk of GERD by altering gastrointestinal function and affecting appetite and dietary habits [45–47]. Smoking is a known risk factor for GERD, as it can lower the pressure of the lower esophageal sphincter and increase gastric acid secretion, thus facilitating acid reflux [48–50]. Therefore, smoking, major depression, and high BMI are more likely to lead to GERD, and our research indicates that GERD is a risk factor for bladder cancer, cervical cancer, lung cancer, lung adenocarcinoma, lung squamous cancer, esophageal cancer, oral cancer, pharyngeal cancer, ovarian cancer, and pancreatic cancer. Thus, GERD may act as a mediating variable, linking BMI, severe depression, and smoking with the incidence of various cancers. This complex intermediary role underscores the pivotal position of GERD in linking lifestyle and mental health factors with the risk of various cancers. Through its significant mediating effect, GERD reveals the potential mechanisms between these factors and cancer occurrence, elucidating how lifestyle habits and psychological states can influence the development of GERD, thereby increasing or reducing the risk of specific cancers. These findings not only help us to understand more deeply the biological basis of these associations but also may provide important guidance for the formulation of prevention and treatment strategies. By integrating strategies aimed at optimizing lifestyle and mental health, we cannot only enhance the management of gastroesophageal reflux disease but also potentially reduce the associated cancer risks.

The investigation employed various sensitivity analyses to enhance the credibility of the conclusions. The consistency among the majority of WM, MR-Egger, and IVW methods further validated the robustness of the study results. Although some outcomes exhibited wide confidence intervals, the overall trend of the associations remained stable. Additionally, the application of the MR-PRESSO method helped identify and eliminate potential outliers, thereby increasing the reliability of the conclusions. Since both the exposure and outcome groups were predominantly of European descent, the likelihood of bias due to population stratification was significantly reduced. Nevertheless, several limitations should be noted. The reliance on genetic data may not fully capture the complexity of the relationship between GERD and cancer risk. Furthermore, the findings, being based primarily on a population of European ancestry, may not be directly applicable to other populations. Although various potential confounders were considered, not all possible factors that might influence the observed associations were included. Additionally, the study did not differentiate between different phenotypes of GERD (such as erosive and non-erosive reflux disease), and varying severity may have different impacts on cancer risk. Future research should explore these aspects to better understand the role of GERD in cancer development. Despite these limitations, the study provides valuable insights into the potential causal relationship between GERD and the risk of various cancers.

## Conclusions

This study provides compelling evidence, significantly revealing a close association between GERD and various types of cancer. This finding further strengthens the notion of considering GERD as an important risk factor in the development of cancer, emphasizing the necessity of incorporating it into comprehensive cancer prevention strategies. Furthermore, this study identifies the critical roles that lifestyle and mental health factors play in regulating the association between GERD and cancer. This presents a novel perspective for future in-depth exploration of this relationship and the development of potential intervention strategies. Based on these findings, future research can further unveil the potential mechanisms underlying the increased cancer risk associated with GERD. A more comprehensive understanding of the mediating roles of BMI, severe depression, and smoking in the relationship between GERD and cancer is especially needed. With the interplay of these variables, clinicians can more accurately predict cancer risk, thereby designing more personalized prevention strategies for patients with GERD.

### Supplementary Information

Additional file 1: Table S1. Overview of data. Table S2. All-selected SNPs in the study. Table S3. Mendelian randomization analysis of GERD association with pan-cancer risk. Table S4. 63 unique potential confounding SNPs. Table S5. Sensitivity analysis for GERD and cancer. Table S6. Outlier analysis and correction of SNPs. Table S7. Reverse MR analysis of cancer on GERD. Table S8. Comparison of effects before and after adjusting variables. Table S9. Mendelian randomization analysis of the major depression, BMI, and smoking initiation association with pan-cancer risk. Table S10. Results from a two-step Mendelian randomization mediation analysis.

## Data Availability

No datasets were generated or analysed during the current study.

## Abbreviations

GERD: Gastroesophageal reflux disease
GWAS: Genome-wide association studies
MR: Mendelian randomization
OR: Odds ratio
CI: Confidence interval
BMI: Body mass index
FDR: False discovery rate
IVW: Inverse variance weighted
WM: Weighted median
IVs: Instrumental variables
SNPs: Single nucleotide polymorphisms
MR-PRESSO: Mendelian Randomization Pleiotropy RESidual Sum and Outlier
TRICL: Transdisciplinary Research in Cancer of the Lung
ILCCO: International Lung Cancer Consortium
IRB: Institutional Review Board
BE: Barrett’s esophagus
MDD: Major depressive disorder
